# What Drives the Usage of Management Tools Supporting Industry 4.0 in Organizations?

**DOI:** 10.3390/s21103512

**Published:** 2021-05-18

**Authors:** Zlatko Nedelko

**Affiliations:** Faculty of Economics and Business, University of Maribor, Razlagova ulica 14, 2000 Maribor, Slovenia; zlatko.nedelko@um.si; Tel.: +386-0222-90-131

**Keywords:** management tools, personal drivers, organizational drivers, utilization, Europe, organizations

## Abstract

The main purpose of this study was to examine how personal and organizational drivers influence the utilization of management tools aimed at supporting organizational working in Industry 4.0 settings. We built our research upon the recognized importance of management tools for organizational working under Industry 4.0 settings and explored the key personal and organizational drivers of management tool usage. Calculations were performed based on the responses of 222 employees working in organizations across Europe. The results revealed that, among personal drivers, a higher level of education leads to significantly higher usage of six sigma, rapid prototyping, outsourcing, customer relationship management, knowledge management, core competencies, and strategic planning. More experienced employees use significantly more six sigma, total quality management, supply chain management, knowledge management, and core competences than their less experienced peers. The impact of organizational drivers is substantially weaker, where only industry shows significant influence, indicating that lean production, six sigma, and supply chain management are used more in manufacturing than in service organizations. Gender, one’s position in the organization, and the organization size do not play a substantial role in management tool usage. Managers should recognize the role of personal and organizational drivers of management tool usage in order to more quickly implement Industry 4.0 principles in organizations.

## 1. Introduction

The role of management tools in supporting organizations working has been widely recognized in the management literature [1,2,3,4,5,6]. Many studies have emphasized the role of single management tools [3,7,8,9,10,11,12,13,14,15], as well as groups of management tools, for supporting organizational working in various circumstances [16,17,18,19].

Recently, along with the need to implement principles of Industry 4.0 in organizations to improve their working and processes [20,21], it has also been emphasized how management tools support the implementation and utilization of Industry 4.0 principles in organizations, as well as support organizations working under Industry 4.0 conditions [22]. There are also theoretical assumptions that certain management tools better support organizational working under Industry 4.0 conditions. In this context, lean management and lean principles are most frequently emphasized, being seen as key building blocks of Industry 4.0 in organizations [23,24,25,26]. Taken together, the existing literature indicates that the most important and promising management tools that are supporting organizations’ work in Industry 4.0 conditions are lean production [24,27,28], rapid prototyping [29], digital transformation [22], etc.

We are familiar with the theoretical assumptions and argumentation [27,30,31,32], as well as few empirical evidence [22], indicating that management tools play a key role in the implementation of Industry 4.0 in organizations, as well as support the work of organizations under Industry 4.0 principles. This leads to a new challenge–what drives the usage of management tools, and which tools are of most interest to organizations working in Industry 4.0 conditions? 

Despite the rising importance of management tools in organizations, from our point of view of supporting organizational working under Industry 4.0 conditions, the research has not kept pace by investigating what drives the usage of management tools. Searching through the literature for drivers of management tool usage returns only partial evidence about potential drivers. This can be found in studies dealing with management tools [1,27,33,34,35,36,37,38,39,40,41], but the aim of these studies is not to reveal the drivers of management tool usage. Those studies are not addressing the drivers directly; rather, the potential drivers are included as “control variables.” Turning to the few studies addressing the drivers of management tools, the literature offers only modest evidence about drivers of management tool usage in organizations. A seminal empirical study by [42] reveals education, position in organization, and number of working years as key drivers of management tool use. To sum up, we can conclude that the most convenient way to consider the drivers of management tools are as control variables in studies of single or a few management tools [42,43], which can present a starting point for deeper research into the drivers of management tool usage. 

Existing studies do not provide sufficient answers to the question of what the key drivers of management tool usage are in the context considered—i.e., Industry 4.0 conditions. To sum up, there is evidence that personal (e.g., age, gender, education) and organizational drivers (e.g., industry or organization size) influence the utilization of management tools that support organizational working under Industry 4.0 conditions.

To consider this gap in the literature, this study provides an initial insight into personal and organizational drivers that influence the usage of management tools that support organizational working under Industry 4.0 conditions. Thus, building upon the empirically confirmed importance of management tools for the implementation of Industry 4.0 in organizations [22], the potential drivers of management tools listed in various studies of management tools [27,33,38,39], and the recognized importance of personal and organizational drivers of management tool usage [42,43], this study investigates how several key personal and organizational drivers influence the usage of several notable management tools in Industry 4.0 conditions. We simultaneously consider the impact of selected personal and organizational drivers on the usage of a single management tool, to obtain more reliable and accurate results. 

The findings of this study will help organizations and their managers to more efficiently manage the usage of management tools in organizations and will enable them to precisely define actions in order to influence single management tool usage and speed up the implementation of Industry 4.0 principles, as well as improve organizational working under Industry 4.0 conditions.

## 2. Theoretical Background and Research Question Development

In this section we first define term management tools and their role in organizations and outline tools that are considered to support organizational work under Industry 4.0 conditions. In the second part of this section, we focus on the presentation of key personal and organizational drivers of management tool usage. We also postulate research questions.

### 2.1. Management Tools

The literature does not offer a uniform definition of the term “management tool.” A simple definition defines management tools as a set of concepts, processes, and exercises aimed at supporting organizations’ operations [44]. Management tools are a way of realizing management ideas and concepts [42]. In that context, the concept may be seen as a comprehensive basis for the consideration of an idea. The next level represents methodology, which is seen as a nexus of methods, rules, and disciplinary postulates. The next level is methods, which are procedures steering the way towards the realization of a given goal. The next level is techniques, which are seen as the manner in which technical details are treated. At the final level are the corresponding management tools, which are considered the way of realizing management ideas. 

To sum up, broadly, management tools can be considered tools that support organizational working at various hierarchical organizational levels, across functional areas and processes in organizations. 

The literature is meager in terms of providing classifications of management tools. Research done by Rigby and Bilodeau, who pioneered the survey of “management tools and trends” [17,45], established the classification of management tools based on usage and satisfaction. In that context, the authors defined the following groups of management tools [6]: (1) Rudimentary implements whose scores for usage and satisfaction are below average. These tools are underdeveloped due to a lack of interest in investing in these tools or the emergent nature of the tools (e.g., RFID, consumer ethnography). (2) Specialty tools that are seldom used but bring about higher levels of satisfaction if they are used in the “right moment.” Such niche tools include, for instance, business process reengineering, mergers, and acquisitions. (3) Blunt instruments that score high on usage but low on satisfaction. An example of such a tool is knowledge management, where organizations and managers strive to acquire knowledge but the satisfaction is often low, as acquiring knowledge is a very sensitive process. (4) Power tools receive high scores for both usage and satisfaction. Those tools are used by many managers with success and bring about great benefits. Those tools are typically strategic planning, customer relationship management, supply chain management, etc. 

Another known classification of management tools is into two groups based on their content [46]: (1) Traditional management tools are most commonly used in organizations. Many of these tools have been evolving for decades and have become crucial for supporting organizational working and behavior. These tools are aimed at supporting customer relationships (e.g., customer relationship management, customer segmentation), the development of an organizational strategy (e.g., strategic planning, mission and vision statements), supply chain management, the optimization of internal processes (e.g., business process reengineering, outsourcing, total quality management), etc. (2) Contemporary management tools, where the main distinguishing characteristic is information technology, as either the tool development is based on and enabled by information technology, or information technology supports the existing idea (e.g., loyalty management, consumer ethnography, rapid prototyping).

The importance of management tool usage has been increasingly recognized by organizations in the last few decades, as is also reflected in the plethora of management tools that are outlined in the most commonly used management books [4,5,47,48] and specific handbooks that aim to serve as guidelines for managers to support their work [18,19]. There is a growing number of management tools that are used to support organizational working and behavior. Especially in the last few years, many management tools based on intensive usage of information technology have emerged, reflecting the current trend of digitalization, such as collaborative innovation, radio-frequency identification, etc. Building on the above “contemporary tools” and the increased usage of information technology in organizations, digitalization and Industry 4.0 have come to the forefront.

### 2.2. Industry 4.0 and Management Tools

Turning to the Industry 4.0 phenomenon, we find many, more or less similar, definitions in the literature, especially from the last couple of years, since the creation of the term Industry 4.0, which was introduced in Germany in 2011 [49,50]. At the heart of Industry 4.0 is the permanent connection of people and things with each other, based on information technology [51]. Moreover, included in that context is enabled collaboration between equipment, people, and other objects in order to manage business processes and value-creating networks [52]. With Industry 4.0-associated technologies, the work within an organization, as well as with suppliers and customers, will be supported [53,54].

In general, we note that there is a lack of surveys that empirically confirm the usage of certain management tools in the Industry 4.0 environment in organizations. Among those, the study done by [22] simultaneously considered the usage of management tools in association with Industry 4.0 and provided evidence about key management tools that support the implementation of Industry 4.0 principles in organizations. That study outlined the following management tools that are associated with Industry 4.0 in organizations, namely, (1) digital transformation, (2) a balanced scorecard, (3) rapid prototyping, (4) radio-frequency identification, (5) six sigma, (6) a mission and vision statement, (7) customer segmentation, and (8) total quality management.

Another stream of studies comprises those focusing on one or a few management tools in association with the Industry 4.0 environment. For instance, very commonly outlined is lean manufacturing or lean principles [27], which are also often emphasized as an important prerequisite and “final step” before Industry 4.0 adoption [55,56]. Moreover, frequently mentioned in association with Industry 4.0 is the six sigma concept [57]. There are also some other tools mentioned in the context of Industry 4.0, such as rapid prototyping [29], supply chain management [53], etc. 

Following the broad consideration of management tools outlined above, a few attempts have been made to outline the key management tools that support organizational working in the Industry 4.0 environment and key characteristics of Industry 4.0 in organizations. The next challenge is to outline the role of the most commonly used management tools in the framework of Industry 4.0 conditions.

Looking from an organizational perspective, the implementation of Industry 4.0 principles brings about many changes to organizational working and behavior. The most notable changes in organizations are as follows. More intensive usage of information technology in organizations across various departments and processes is reflected in the usage of several management tools that are relatively new and based on information technology, such as radio-frequency identification, which enables fast product data management via tags [58]; rapid prototyping, which allows for a shorter development cycle for products [29]; and shared service centers, which enable the usage of the service by several users in an organization, as well as outside the organization [17,37]. 

Next, we take a look at management tools that are still supported by information technology but are of special interest to organizations. A set of management tools is designed for the execution of business processes in organizations [59], where the management tools support business process reengineering, which is necessary due to the implementation of Industry 4.0 principles, as well as other changes to processes and functions. Those management tools are lean production [23,26,27,56], six sigma [32,33], and total quality management [60], which improve the processes and the overall organizational working. Additionally, outsourcing can also be considered important in the framework of optimizing organizational working [61].

Implementation of the Industry 4.0 principles also enhances interaction with suppliers and customers, where the utilization of modern information technology will advance current methods of collaboration between organizations and their suppliers and customers [53]. In that context, supply chain management tools and customer relationship management tools are crucial.

Changes due to the Industry 4.0 principles’ implementation in organizations are also part of the field of human resource (HR) management [62,63]. At the forefront is interest in improving employees’ personal and professional competence to work in the Industry 4.0 environment [63,64,65]. Knowledge management will have an important role to play [66], as well as tools supporting the development of core competencies to align the existing competencies of employees with those needed in an Industry 4.0 environment [67]. 

Last but not least, strategic planning also has an important role [21,43], as it is an important first step toward Industry 4.0. Strategic planning thus must set the direction of the organization towards Industry 4.0 [68]. This is especially important in organizations having a lower level of readiness for Industry 4.0 and still needing to establish the goals associated with Industry 4.0 adoption and implementation.

To sum up, we are focusing on the following management tools, which aim to support the work of organizations under Industry 4.0 conditions, namely, (1) lean principles, (2) six sigma, (3) rapid prototyping, (4) radio-frequency identification, (5) shared service centers, (6) total quality management, (7) outsourcing, (8) supply chain management, (9) customer relationship management, (10) knowledge management, (11) core competencies, and (12) strategic planning.

At this point, now that we have defined the set of management tools that support the work and behavior of organizations in Industry 4.0 conditions, the key question capturing our attention is what drives the usage of the outlined management tools. This will be the focus of the following subsection.

### 2.3. Drivers of Management Tool Usage

As the level of management tool utilization in organizations is increasing, and management tools are important for supporting organizational working and behavior, especially in the Industry 4.0 conditions outlined above, this study seeks to address what drives the usage of management tools, aimed at supporting organizational working and behavior under Industry 4.0 conditions. 

Exploring the literature offers meager direct evidence of the drivers of management tool usage. There is no comprehensive overview of the drivers of management tool usage, especially those management tools that have been at the forefront of interest since the advent of Industry 4.0. 

There might be some evidence of possible drivers of management tools if we consider studies dealing with management tools. As the aims of these studies are not to reveal the drivers of management tool usage, those studies may be useful if “control variables” are included in the surveys. Those variables may indicate whether there is an association between the usage of the management tools considered and specific control variable(s). In order to get insight into the potential drivers of management tools, we focused on a few frequently cited studies dealing with single management tools. This was to give us insight into the possible drivers of management tool usage and to provide the building blocks for the determination of the drivers of management tool usage. In Table 1, we present an overview of the possible drivers of management tool usage, extracted from studies of management tools, where the researchers consider various potential drivers of management tool usage.

Table 1 provides insight into the variables (i.e., control and demographic) that are considered along with the selected management tool in each study. The outlined studies above do not explicitly emphasize the considered variables as drivers of management tool usage. An exception is [42], which focused on three drivers of management tool usage. 

### 2.4. Research Questions 

Based on our knowledge of the most common variables that are included in single studies of management tools, we next outline a few potential personal and organizational drivers of management tool usage. 

#### 2.4.1. Personal Drivers

We start with a group of potential personal drivers of management tool usage, extracting the mentioned personal drivers from studies of management tools in various contexts, as outlined in Table 1. Based on [42], which directly studied the drivers of management tool usage, we extracted the education and position of the employee in an organization as drivers of management tool usage. Turning to management studies that consider several management tools simultaneously, we zeroed in on a report [43] on education, position, and number of working years as drivers of management tool usage. 

A potential set of personal drivers can be confirmed by the personal demographic variables used in other business studies. Of the personal demographic variables used, often as control variables, the most frequently outlined are age, gender, education, number of working years, and the position of employees in the organization [89,90,91] (see Table 1 for details of the selection of personal drivers of management tool usage). In the next few paragraphs, we look closely at these potential personal drivers of management tool usage.

Looking from a general perspective, age has been emphasized as an important demographic and control variable in many business studies where employees are surveyed for their opinions [36,69,92,93]. For instance, [94] reported that the use of computers is highly stratified by age, and the usage of computers decreases with age. Olson et al. [95] reported that younger adults use a broader spectrum of technologies than older adults, and also stressed that age-related differences in the usage of technology depend on the technology itself. Naturally, we would conclude that younger employees will use management tools more, but one could also argue that management tools would be used more by older employees as they have more experience. Inversely, when it comes to the newest management tools, especially those based on information technology, younger employees may have the advantage if they learned about them in the course of their education. Due to the inconclusiveness of the findings and the usage of age as a control variable in many studies including management tools [36,38,69], we postulate the following research question (RQ):

RQ1: How does age influence the usage of management tools in organizations?

Gender is the next typical demographic variable in business studies and has been frequently emphasized as playing a decisive role in the context of studying certain phenomena. For instance, [96] examined the difference in willingness to engage in unethical business behavior between male and female respondents, while [97] examined entrepreneurs’ gender-specific personal characteristics. Inversely, studies of adolescents do not reveal any significant differences between males and females regarding information technology literacy and confidence [98]. Similarly, a study of the influence of gender on new technology adoption and the use of mobile commerce did not reveal any gender-related differences in the usage of new technology [99]. Turning to the studies considering management tools [1,36,38,70,73], gender showed some associations with the studied phenomena in the descriptive statistics. Therefore, we postulated the following RQ:

RQ2: How does gender influence the usage of management tools in organizations?

Next, very frequently considered in business studies is education level [90,91]. Turning to the studies of management tools from various perspectives, it is evident that many of the studies outlined in Table 1 consider level of education as a variable that has a potential impact on the use of the management tools studied [1,36,38,40,43,69,73,77], though further examination of the role of education in those studies is limited. In terms of education, with the increase in the level of education, the probability of knowing and using these management tools later on in the working environment increases substantially. Two surveys of management tool usage found a positive association between the level of education and the usage of management tools, implying that those employees with a higher level of education use management tools to a greater extent [42,43]. An exception is [42], where a higher usage of core competencies was reported by those with lower levels of education. Secondary school education does not include many management tools in the curriculum, while the curricula of business schools have many management tools integrated into the content. For instance, classes include strategic planning, CRM, total quality management, etc. Despite a relatively uniform association between the usage of management tools and the level of education, we still allow for the possibility that management tools not previously considered in research can lead to different results in terms of usage. Therefore, we postulate RQ as follows:

RQ3: How does the level of education influence the usage of management tools in organizations?

The position in an organization is another variable frequently examined in business studies in general [90,100], as well as in studies examining management tools from various standpoints [1,35,37,39,72,80,83,84]. Two existing studies about drivers of management tool usage reported similar results [42,43], with all the considered management tools used more at higher organizational levels. Despite this uniformity of results, we need to consider that the impact of position on management tool usage may be more complex. Therefore, we cannot simply postulate that, at a higher organizational level, the usage of management tools will be higher. We argue that position may also substantially influence the usage of management tools, as the nature of the work at different organizational levels is different. For instance, we need to distinguish between management tools that support work at different organizational levels. For instance, CRM is aimed to support work especially at the nonmanagerial level, while for strategic planning is expected that the utilization of management tools will be higher at higher organizational levels as strategic planning is in the domain of top management [101]. Therefore, we cannot, for instance, automatically assume that CRM will also be more used by managerial working positions. In line with the reasoning outlined above, we postulate the following RQ:

RQ4: How does the position in the organization influence the usage of management tools?

#### 2.4.2. Organizational Drivers

There are no studies that delineate the impact of typical organizational drivers on management tool usage. In terms of organizational drivers that may influence the utilization of management tools, the selection is narrower compared to personal drivers, but it is clear that the studies that emphasize management tools [1,27,33,35,39,76,81] (see also Table 1) frequently consider organizational size as important in their surveys. This is a clear indication that organizational size is considered to have an impact on the studied management tools. 

Dabic et al. [43] highlighted organizational size as one of the drivers of management tool usage and reported mixed results as to how the organizational size influences it; their study reported a significant association with mission and vision statements, while for other management tools in the analysis, the associations were not significant. 

Looking beyond the mentioned study, we can see that larger organizations use management tools to a greater extent than smaller ones for several reasons. For instance, more financial and human resources are available to support the usage of a number of management tools in larger organizations. Based on the above considerations, we postulate the following RQ:

RQ5: How does organizational size influence the usage of management tools in organizations?

A final driver of management tool usage considered in this paper is the industry of the organization, which, again, is often considered in research dealing with management tools. In those studies, researchers consider the industry as an important factor and often include organizations and participants from various industries in a study [35,37,39,71,72,74,82], distinguishing between manufacturing and the service industry [82,88], or focus on a specific industry, such as service [1,85,86] or manufacturing [33,78,83,87]. However, the organization’s industry is frequently considered as a control variable when studying select phenomena in business research; one of the few studies of management tools emphasized the organization’s industry as a driver of management tool usage [42,43]. For instance, [102] also reported that management tools are more frequently used in manufacturing than in service organizations. In light of the lack of studies on the impact of organizations’ industry on management tool usage, we postulate the following RQ:

RQ6: How does the industry of an organization influence its usage of management tools?

## 3. Methods

### 3.1. Instruments Used

We used a modified version of the questionnaire about knowledge, usage, and satisfaction with management tools in organizations used in prior research about management tools [16,43,46]. After adding some new management tools, the current version includes 33 management tools. The questionnaire has three parts. Part 1 gathers demographic data about respondents and their organizations. Part 2 gathers general information about the use and knowledge of management tools in organizations. Part 3 provides a matrix in which respondents assess their usage of management tools, knowledge of management tools, and satisfaction with tool usage, for each of the 33 management tools listed.

### 3.2. Sample and Procedure

The survey was conducted in 2019 among employees in organizations across Europe. Based on random sampling of organizations, we collected approximately 2000 direct email addresses of employees in organizations where the focus was on managerial positions. We sent the link to the online questionnaire to selected addresses. We received 222 usable questionnaires from respondents in different European countries, indicating an 11.1% response rate.

In terms of the sample characteristics, our convenience sample included 53.8% males and 46.2% females. The average age of respondents was 32.56 years. Regarding education, 33.8% of participants finished high school, 36.9% have a bachelor’s degree, 25.7% have a master’s degree, and 3.7% have a PhD. Regarding the position of respondents in organizations, 23.4% of respondents are working at nonmanagerial positions. In terms of organizational size, 44.4% of respondents work in organizations with fewer than 100 employees, 29.4% in organizations having between 100 and 1000 employees, and 26.2% in organizations with more than 1000 employees. In terms of organizations’ industry, 14.9% are involved in manufacturing, while 85.1% are in the service industry. 

### 3.3. Measures

In line with the aims of this study, the following measures were used in calculations. Personal and organizational drivers of management tool usage were extracted from the first part of the questionnaire, while the usage of management tools was extracted from the third part of the questionnaire.

Personal drivers—the personal drivers of management tool usage were assessed as follows. Age: respondents put in their age. Gender: respondents selected their gender, male or female. Education: respondents chose the highest achieved education level, ranging from high school through a PhD. Position in organizations: respondents selected their position in the organization, ranging from nonmanagerial position to top manager. 

Organizational drivers—the two drivers considered were measured as follows. Industry of organization: respondents had to those their industry from the NACE classification, offering 22 possible industries. As this variable cannot be considered as an interval, 21 dummy variables will be needed to enter this variable into the regression analysis. For instance, in [82], a study of total quality management, and [88], a study of strategic planning in SMEs, the authors distinguished between the manufacturing and service industries. Therefore, we also distinguished between manufacturing and service [82,88,103] and merged the industry variable as a dichotomous variable with two categories, namely, manufacturing and service. In the manufacturing category, we merged all industries from A to C according to the NACE classification, while we placed in the service category all remaining industries, from D to the end. In terms of organizational size, respondents entered the approximate number of employees in their organizations. We classified their organization as a small and medium organization with fewer than 100 employees, a medium-sized business with between 100 and 1000 employees, or a large business with more than 1000 employees, according to the NAICS classification [104]. 

*Management tool utilization*—the respondents assessed their level of usage of each of the 33 management tools listed, by using an interval scale ranging from “I always use (1)” to “I never use (7)”. Respondents assessed each tool by selecting one value.

### 3.4. Research Design and Analysis

In keeping with the purpose of this research, we first outline elements of descriptive statistics, including mean values, standard deviations, and zero-ordered correlations between the variables of interest in this study. Second, we conducted a set of hierarchical regression analyses to examine the impact of personal and organizational drivers on management tool usage. In the first step, we entered personal drivers of management tool usage, namely age, gender, education, and position (model 1 (M1)), while in the second step we added organizational drivers of management tool usage, namely organizational size and industry (model 2 (M2)). We repeated the hierarchical regression analysis for all 12 management tools included in the survey.

We calculated collinearity statistics for each of 12 hierarchical regression analyses, between the drivers of management tools and usage of management tools. VIF and tolerance values were all in the acceptable range suggested by [105]. Multicollinearity is thus not a problem in this study. 

As the sample for this study consists of answers from various European countries, we adopted the distinction between well-developed and former transition economies (i.e., catching-up economies) [16,92,106,107,108,109]. Accordingly, we formed two groups, namely: (1) well-developed economies, including employees from Germany, Spain, Italy, France, and the United Kingdom and (2) former transition economies, including employees from Hungary, Serbia, Albania, Turkey, and the Czech Republic. We named this variable “country.” We did not include in the analysis the number of working years or experience of employees as one of the possible personal drivers of management tool usage [42], since a very strong correlation exists between the age of the respondent and their number of working years (β = 0.90; *p* < 0.001). As there is a perfect correlation between age and work experience, we consider age as an adequate variable to explain our results. The inclusion of number of working years in the analysis could trigger some multicollinearity issues. 

## 4. Results

The mean values, standard deviations, and correlations between variables of the interest are outlined in Table 2.

Some of the associations from Table 2 are noteworthy. First, the level of education has the strongest influence on management tool usage in organizations, followed by the respondent’s age and position in the organization. Second, only a few organizational drivers have a significant impact on management tool usage. Third, some associations between the usage of management tools are moderately statistically significant. 

As we have responses from employees in 10 countries, and we distinguish between well-developed and former transition economies, differences may be seen between employees from different countries in terms of management tool utilization. Table 2 reveals a weak significant impact of country on management tool utilization in two instances, namely knowledge management (β = −0.18; *p* < 0.5) and core competencies (β = 0.15; *p* < 0.5). Results from an independent samples t-test also confirmed differences in the usage of knowledge management (t = 2.59, *p* < 0.05) and core competences (t = −2.16, *p* < 0.05) between well-developed and former transition economies. Knowledge management is used more in former transition economies (mean value = 3.10) and less in well-developed economies (3.77), while core competencies are used more in well-developed economies (3.50) than in former transition economies (4.08). As the differences are not substantial, we focus on drivers of management tools; the two emphasized management tools are of less importance for organizational working in an Industry 4.0 conditions, so we do not include “country as variable” in the further analysis. 

Next, we will outline the impact of personal (model 1) and organizational drivers (model 2) on management tool utilization. The results for the impact of selected drivers on the usage of lean principles, six sigma, rapid prototyping, radio-frequency identification, shared service centers, and total quality management are presented in Table 3. The results for outsourcing, supply chain management, customer relationship management, knowledge management, core competencies, and strategic planning are outlined in Table 4.

Considering the results in Table 3 and Table 4 through the prism of the postulated RQs, we note that, for five out of the 12 management tools considered, the age of the respondent has a significant impact on management tool utilization. The impact is uniform in all five instances, showing that older employees use six sigma, total quality management, supply chain management, knowledge management, and core competences to a higher extent than their younger peers. The impact of the respondent’s highest achieved educational level on the usage of management tools is significant for seven out of 12 management tools. Again, the impact is uniform, and it is evident that employees with the highest achieved education use six sigma, rapid prototyping, outsourcing, customer relationship management, knowledge management, and core competences to a higher extent than those with a lower level of education. In terms of the impact of gender and the employee’s position in the organization, these variables did not have a significant impact on usage for any of the 12 management tools considered.

Turning to the two organizational drivers, the impact of organizational size on usage of the 12 management tools considered is nonsignificant, while the impact of industry is significant for three out of 12, indicating that lean production, six sigma, and supply chain management are more frequently used in manufacturing organizations than in service organizations.

## 5. Discussion

The impact of the considered personal and organizational drivers on management tools supporting organizational working under Industry 4.0 conditions was examined in this study. At first glance, it is evident that personal drivers have a substantial impact on the usage of management tools, aiming to support the working of organizations in the Industry 4.0 environment, as compared to the limited impact of organizational drivers. 

Among the personal drivers considered, the strongest impact is associated with the respondent’s level of education, indicating that employees with a higher level of education more frequently use management tools than their peers with a lower educational level, corroborating the results of prior surveys [42,43]. Those management tools are six sigma, rapid prototyping, outsourcing, customer relationship management, knowledge management, core competencies, and strategic planning. Looking for reasons for the dominant impact of education on the usage of management tools, we note that business schools have integrated management tools into their curricula. Therefore, students become aware of them and continue to use them when they enter organizations, especially those associated with the Industry 4.0 environment.

In terms of the strength of the impact, the age of employees was the second most important variable after education. As outlined above, due to the perfect correlation between the number of working years and age, it becomes evident that the work experience of employees plays a major role in management tool usage. Thus, those employees with more experience use management tools to a greater extent than those with less work experience. This sounds reasonable, as those with more experience are more familiar with the organizational settings, processes, procedures, and in general with all the tools used in the organization. 

Among the personal drivers in this study, the position of employees does not play a substantial role in management tool usage, which is contrary to what was found in previous studies, which reported that management tools are used more at higher organizational levels [42,43]. The reason for the difference may lie in the structure of the sample, as in our study it reflects the ratio between manufacturing and service organizations, as well as the fact that the sample comprises employees from across Europe. Prior studies considered the usage of management tools in Slovenia and Croatia, two former transition economies sharing some specifics in their organizational settings due to the transitional background [107,110]. Thus, we may argue that the position does not have more influence on the usage of management tools, as they are used across the entire organization. What is surprising is that the position in the organization is often considered in studies of management tools as potentially decisive for the studied phenomena (i.e., considered management tool), but many studies then do not consider the respondent’s position in the organization in a further analysis—i.e., in basic descriptive statistics or beyond [35,39,84,111]. 

The last of the personal drivers of management tool usage, gender, had no significant impact on the usage of the considered management tools that aim to support organizational working under Industry 4.0 conditions. This is in line with studies reporting that gender does not have an influence on the studied phenomena [98,99]. The nonsignificant role of gender in management tool usage also supports the notion that males and females are “equal in terms of management tool usage”, showing progress toward reducing the workplace gender gap [112].

Turning to the organizational drivers of management tool usage considered, namely, the organizational size and industry, it is evident that they both play a relatively minor role in determining the usage of management tools, compared to personal drivers. As industry is often considered in studies of single management tools—for instance, comparing manufacturing and service organizations [82,88]—one could expect a more substantial influence of industry on the usage of the considered management tools in this survey. However, industry only has a significant impact on the utilization of lean principles, six sigma, and supply chain management, where it is evident that lean practices are used significantly more by manufacturing than service organizations. Treven, Uršič, and Rashad [1] reported that supply chain management—considered as enterprises’ operations in supply chains—is used more in manufacturing than in service organizations. Similarly, [33] reported that six sigma is used more in manufacturing than in the academic sector, especially six sigma “black and master black belt.” All these facts confirm the finding from prior studies that lean principles have their roots in manufacturing [113] and were dominantly implemented in manufacturing organizations [71,114]. Moreover, the supply chain management literature puts much more emphasis on manufacturing organizations, as focal organizations [115], than on service organizations. 

Turning to the impact of organizational size, in our study, there was no substantial impact of organizational size on management tool usage. This might be a bit surprising, as organizational size is often considered in studies of single management tools [33,35,81]. For instance, [73] examined how the business orientation of manufacturing enterprises determines the utilization of lean production. Correlations in this study reveal that, in a sample of Arabian Peninsula organizations, lean production is used substantially more than in smaller organizations. The authors also reported that organizational size does not have a significant impact on the usage of lean production in Western and Central Europe. Thus, we can argue that our finding of a nonsignificant association between organizational size and the usage of lean production in the European sample echoes the results of the aforementioned study. 

The sample in this survey included employees from 10 different European countries. It would be expected that differences in management tools would occur [16,17], yet there are only two instances of significant differences in the utilization of management tools between employees from well-developed and former transition economies. We can argue that the higher utilization of knowledge management in former transition economies than in well-developed economies is attributed to the need to increase the level of knowledge of employees, which is often at a lower level, comparatively, and may have been acquired from headquarters abroad. On the other hand, core competencies are used more in well-developed economies than in former transition economies. This indicates that organizations in well-developed economies put a lot more emphasis on the “human factor,” in this case, on strengthening employees’ competencies, than those in former transition economies, where striving for the optimization of processes is still an important issue [16].

Last but not least, the associations between management tools are also noteworthy. Several moderate and significant associations between the considered management tools indicate their complementary nature and reveal that management tools are simultaneously used in organizations to support organizational working across all processes, functional areas, and hierarchical levels. 

### 5.1. Theoretical Implications

The main theoretical implications of this study are as follows. The study’s findings set the stage for a further examination of the drivers of management tool usage, as we determined the strength of the impact of several personal and organizational drivers of management tool usage, while the current literature dedicates little attention to them, especially in the form of a simultaneous consideration of several management tools. A moderate and significant association between the usage of management tools also implies that research on management tools needs to consider a broader range of management tools due to their inter-relatedness and the possible spillover effect that simultaneous use may produce.

### 5.2. Implications for Practice

The most notable practical implications of this study are as follows. Managers in organizations need to recognize the role of key personal and organizational drivers for management tool usage. Thus, if the management in organizations want to speed up the implementation of Industry 4.0 practices, this can be done via boosting the usage of management tools [22]. As this study shows, managers also need to give adequate attention to single drivers of management tool usage and tailor actions accordingly to increase or decrease the usage of single management tools. 

The dominant impact of personal drivers (over organizational drivers) on the usage of management tools supporting organizational working in an Industry 4.0 setting implies that organizations and their managers can influence the usage of management tools. For instance, among older employees and those with higher education, we see significantly more use of tools than among younger employees and those with lower education. Organizations’ strategies to bridge this gap could include the following: (1) Conducting in-service training for employees to increase their knowledge about management tools as well as to ensure the availability of the tools in organizations. (2) Hiring practices may also be changed in such a way as to add questions regarding knowledge about specific management tools and the new candidate’s level of familiarity with them. (3) Coaching to transfer knowledge from employees with more work experience to those in the early stages of their career in order to boost the usage of management tools. Furthermore, the training of newcomers in organizations should be designed in such a way that newcomers will be made familiar with the management tools used by their older peers in the organization.

This might seem paradoxical as younger employees use fewer management tools, even though, frequently, they are well educated when they enter the organization. Thus, one may argue that younger employees are not using management tools as frequently as their older peers, as they are not yet in managerial positions. However, this assumption is not supported by our results, as the position in the organization is not a substantial driver of management tool usage. The explanation may lie in the age of the employees and their amount of work experience. As age and number of working years were perfectly correlated in this study, we can argue that work experience has a substantial impact on management tool usage.

Lean principles are used much more in manufacturing organizations, implying that managers in service organizations need to put more effort into increasing the use of lean principles, which are primarily used in manufacturing organizations, to reap the benefits of removing waste from every “corner of the organization” [116]. Similarly, supply chain management, used in service organizations, presents an important opportunity to improve the collaboration between organizations and their partners. 

The determining role of level of education achieved in management tool usage has implications for business schools as well, as they need to ensure that the newest management tools will be introduced to students, who will later on use them in organizations. In that way, the education variable also makes an important contribution to fostering the usage of management tools that support organizations working in Industry 4.0 conditions. 

### 5.3. Limitations

The most significant limitations of the study are as follows. First, a minor limitation is the self-assessment approach used for obtaining respondents’ answers about their usage of management tools in organizations, although this way of gathering responses in business surveys is acceptable and widely used [89,92]. Second, a limited number of management tools is considered. Additional management tools that are closely associated with Industry 4.0 practices, such as digital transformation, can be added to the research in the future [22]. Third, this study did not examine the link between the usage of management tools and the Industry 4.0 working environment. Rather, our selection of management tools was based on theoretical assumptions about how management may support organizational working under Industry 4.0 conditions, or on prior studies [22]. Fourth, we considered organizational industry by distinguishing between organizations involved in manufacturing and service organizations [82,88], in order to reduce the number of dummy variables; had we used the NACE classification, we would have had to include 22 different industries [103]. Fifth, despite having in the sample employees’ responses from different European countries, we did not include this variable in the hierarchical regression analysis, as the focus is on drivers of management tools, not on the comparison between well-developed and former transition economies. 

### 5.4. Future Research Directions

Several future research directions can be outlined. It would be beneficial to examine the associations between key personal and organizational drivers of management tools, by comparing well-developed vs. former transition economies, as the results may vary across economies with different developmental levels [16]. Next, drivers of management tools can also be examined in a broader international context [17,44], by comparing samples worldwide. The examination of drivers of management tools can also be done through the lenses of various industries, to identify whether there are differences between them. Adding contemporary tools to the tools list in the survey will enhance the accuracy of prediction, showing how management tools support organizational working in Industry 4.0 conditions. Finally, measuring the organizations’ level of Industry 4.0 implementation will sharpen the entire picture. 

## Figures and Tables

**Table 1:** Potential drivers of management tool usage ^a^.

Management Tool	Potential Drivers of Management Tools	Reference
LP	Organizational size	
	Age, education, work experience	[69]
	Gender	[70]
	Industry	[71]
	Industry, geographical area, company size, participant position	[72]
	Age, gender, education, position, organizational size, region	[73]
SS	Industry	[74]
	Industry, organizational size	[33,75]
	Age, work experience, education	[69]
	Organizational size	[76]
	Education level	[77]
RP	Organizational size	[34]
	Industry	[78]
RFID	Position in organization, industry of organization, organizational size	[35,79]
	Age, gender, education	[36]
SSC	Position in organization, organizational size	[80]
	Organizational size	[81]
	Industry, organizational size, position in organization	[37]
TQM	Industry	[82]
	Position in organization, organizational size, industry	[83]
	Position in organization, organizational size	[84]
	Age, gender, education, work experience	[38]
	Age, gender, education, position, number of working years, organizational size, industry	[43]
OUT	Position in organization, organizational size, industry	[39]
	Industry, geographical location	[85]
	Industry	[86]
	Organizational size, industry	[87]
SCM	Education level, organizational size, industry	[40]
	Age, gender, education, position, number of working years, organizational size, industry	[43]
	Gender, education, number of working years, position in organization, organizational size, industry	[1]
CRM	Education, number of working years, position in organization	
KM	Age, gender, education, position, number of working years, organizational size, industry	[43]
CC	Industry	[41]
	Education, number of working years, position in organization	[42]
SP	Organizational size, industry	[88]

^a^ LP—lean principles; SS—six sigma; RP—rapid prototyping; RFID—radio-frequency identification; SSC—shared service centers; TQM—total quality management; OUT—outsourcing; SCM—supply chain management; CRM—customer relationship management; KM—knowledge management; CC—core competencies; SP—strategic planning.

**Table 2:** Means, standard deviations, and correlations ^a^.

Variable	M	SD	1	2	3	4	5	6	7	8	9	10	11	12	13	14	15	16	17	18
1. Age	32.56	10.46	1																	
2. Gender	1.46	0.50	−0.14 *	1																
3. Education	2.91	1.00	−0.08	−0.02	1															
4. Position	1.77	0.42	0.18 *	0.00	0.14 *	1														
5. Organizational size	1.82	0.82	−0.02	0.05	0.12	−0.16 *	1													
6. Industry	1.85	0.36	−0.02	0.13 *	−0.04	0.07	−0.32 **	1												
7. Country	1.62	0.49	0.02	−0.02	0.15 *	0.07	−0.14 *	0.14 *	1											
8. LP	4.43	1.96	−0.04	0.09	0.01	−0.04	−0.05	0.30 ***	−0.05	1										
9. SS	5.11	1.84	−0.22 **	0.02	−0.14	0.03	−0.14	0.25 **	0.10	0.36 ***	1									
10. RP	5.20	1.81	−0.11	0.13	−0.19 *	0.07	−0.15	0.13	0.07	0.33 ***	0.48 ***	1								
11. RFID	5.09	1.86	−0.13	−0.04	−0.13	−0.11	0.07	−0.05	0.02	0.13	0.34 ***	0.29 ***	1							
12. SSC	4.50	1.85	−0.12	−0.06	−0.05	0.00	−0.08	−0.04	−0.08	0.26 ***	0.30 ***	0.27 ***	0.37 ***	1						
13. TQM	3.53	1.91	−0.21 **	0.01	−0.05	−0.17 *	−0.02	0.05	−0.09	0.33 ***	0.24 **	0.06	0.08	0.22 **	1					
14. OUT	4.51	1.91	−0.05	0.05	−0.18 **	0.04	−0.16 *	0.08	−0.11	0.31 ***	0.40 ***	0.44 ***	0.22 **	0.46 ***	0.25 ***	1				
15. SCM	3.84	2.05	−0.16 *	0.01	−0.14 *	−0.14	0.06	0.13	−0.11	0.37 ***	0.19 *	0.17 *	0.05	0.21 **	0.41 ***	0.20 **	1			
16. CRM	3.23	1.97	−0.06	−0.02	−0.13	−0.03	0.04	0.05	−0.08	0.27 ***	0.17 *	0.11	0.21 **	0.26 ***	0.33 ***	0.15 *	0.24 **	1		
17. KM	3.34	1.82	−0.15	−0.01	−0.21 **	−0.13	0.06	−0.09	−0.18 *	0.03	0.06	−0.03	0.14	0.29 ***	0.28 ***	−0.03	0.34 ***	0.22 **	1	
18. CC	3.87	1.90	−0.15 *	−0.05	−0.19 **	0.09	−0.17 *	0.03	0.15 *	0.24 **	0.42 ***	0.34 ***	0.22 **	0.40 ***	0.21 **	0.35 ***	0.21 **	0.37 ***	0.19 **	1
19. SP	3.19	1.79	−0.13	−0.02	−0.16 *	−0.18 **	0.05	0.07	−0.06	0.09	0.16 *	0.12	0.04	0.25 **	0.31 ***	0.13	0.30 ***	0.30 ***	0.39 ***	0.32 ***

^a^ * *p* < 0.05; ** *p* < 0.01; *** *p* < 0.001; LP—lean principles; SS—six sigma; RP—rapid prototyping; RFID—radio-frequency identification; SSC—shared service centers; TQM—total quality management; OUT—outsourcing; SCM—supply chain management; CRM—customer relationship management; KM—knowledge management; CC—core competencies; SP—strategic planning.

**Table 3:** Hierarchical regression results ^a^.

	LP		SS		RP		RFID		SSC		TQM	
	M1	M2	M1	M2	M1	M2	M1	M2	M1	M2	M1	M2
Block 1: Personal drivers												
Age	−0.03	−0.02	−0.24 **	−0.24 **	−0.12	−0.11	−0.12	−0.12	−0.14	−0.14	−0.21 **	−0.21 **
Gender	0.09	0.05	0.01	−0.02	0.11	0.11	−0.06	−0.06	−0.09	−0.07	−0.02	−0.03
Education	0.16	0.02	−0.17 *	−0.15 *	−0.20 **	−0.19 **	−0.14	−0.15	−0.06	−0.05	−0.05	−0.05
Position	−0.03	−0.06	0.10	0.08	0.13	0.11	−0.08	−0.07	0.04	0.03	−0.11	−12
Block 2: Organizational drivers												
Organizational size		0.03		−0.03		−0.08		0.06		−0.08		−0.01
Industry		0.30 ***		0.24 **		0.08		−0.01		−0.05		0.06
*N*	183	183	183	183	170	170	183	183	189	189	203	203
*R^2^*	0.01	0.07	0.08	0.14	0.08	0.09	0.05	0.05	0.03	0.03	0.07	0.07
Model F	0.52	3.12 **	3.91 **	4.9 3***	3.47 **	2.77 *	2.21	1.58	1.28	1.03	3.45 **	2.41 *

^a^ Standardized regression coefficients are shown. * p < 0.05; ** *p* < 0.01; *** *p* < 0.001.

**Table 4:** Hierarchical regression results ^a^.

	OUT		SCM		CRM		KM		CC		SP	
	M 1	M2	M 1	M2	M 1	M2	M 1	M2	M 1	M2	M 1	M2
*Block 1: Personal drivers*												
Age	−0.07	−0.07	−0.15 *	−0.16 *	−0.07	−0.08	−0.17 *	−0.17 *	−0.21 **	−0.21 **	−0.11	−0.12
Gender	0.02	0.03	−0.01	−0.05	−0.01	0.02	−0.05	−0.04	−0.08	−0.10	−0.01	−0.03
Education	−0.20 **	−0.18 *	−0.13	−0.13	−0.17 *	−0.18 *	−0.21 **	−0.22 **	−0.23 **	−0.21 **	−0.16 *	−0.18 *
Position	0.09	0.06	−0.07	−0.07	0.01	0.02	−0.06	−0.04	0.17 *	0.14	−0.13	−0.13
*Block 2: Organizational drivers*												
Organizational size		−0.13		0.12		0.08		0.06		−0.08		0.09
Industry		0.04		0.18 *		0.06		−0.07		0.13		0.10
*N*	192	192	192	192	210	210	204	204	199	199	210	210
*R^2^*	0.04	0.07	0.05	0.08	0.03	0.03	0.08	0.09	0.10	0.13	0.07	0.08
Model F	2.17	2.13	2.57 *	2.80 *	1.58	4.99	4.18 **	3.17 **	5.23 ***	4.56 ***	3.72 **	2.91 *

^a^ Standardized regression coefficients are shown. * p < 0.05; ** *p* < 0.01; *** *p* < 0.001.

## Data Availability

Not applicable.
